# Medical Researches on the Effects of Iodine in Bronchocele, Paralysis, Chorea, Scrofula, White Swellings, &c.

**Published:** 1826-01

**Authors:** 


### MEDICO-CHIRURGICAL

### REVIEW.

Vol. VIII.] Analytical Series. [No. 23.

"Nec tibi quid liceat sed quid fecisse decebit
"Occurrat, mentemque domat respectus honesti."

Vol. IV.] JANUARY 1, 1826. [No. 7.

[NEW SERIES.]

### I.

*Medical Researches on the Effects of Iodine in Bronchocele, Paralysis, Chorea, Scrofula, Fistula Lachrymalis, Deafness, Dysphagia, White Swelling, and Distortions of the Spine.* By Alexander Manson, M.D. Physician to the General Hospital, and St. Mary's Hospital and Dispensary, Nottingham. Octavo, pp. 454. London, August, 1825.

THERE can be little doubt that the spirit of enquiry, the taste for experimenting, and the progress of chemistry will, even in our own time, open out immense discoveries in the healing art. When we survey the strange, the unlooked for, and the manifold operations of physical agents on the human frame and its functions, we must be convinced that there is every probability in the expectation of almost innumerable other agencies to be yet detected in the elements and combinations of the animal, vegetable, and mineral substances around us. Who could, *à priori*, imagine, that spurred rye would act specifically on the moving fibres of the uterus? Who would suppose, before trial was made, that a simple and elementary substance, which modern chemistry has denominated, from its colour rather than its qualities, Iodine, would have the *specific* effect of causing absorption of the thyroid gland, when enlarged from the operation of some other substance in nature with which we are not yet well acquainted? Who would believe, 10 or 20 years ago, that of 60 grains of cinchona 59 might be thrown away, and the remaining unit or grain would still be equal, perhaps superior, in medicinal agency, to the whole of the original quantity? Yet, such is the fact, as proved, not merely by chemistry, but by wide therapeutic experience. Chemistry, by enabling us to get at the elementary or active principles of vegetable and mineral substances, promises to do wonders for the practice of medicine. There may be thousands of substances yet concealed in nature, which are possessed of more wonderful specific properties than any we have yet discovered, large as the list is of medicinal agents. We have every reason, therefore, to look forward with hope, and even confidence, to the future.

The subject of the volume before us has, of late years, excited much attention, in consequence of its unequalled power in removing one of the most disgusting, and sometimes a very dangerous deformity, as it affects more especially the female sex. There is no remedy so powerful in reducing bronchocele as iodine:—and if it possess the power of acting on one gland, it may have similar power on other glands. Analogy, however, is of little use in pharmacology. Every thing must be submitted to trial—and in this consists the great merit of the present publication, since it is a series of appeals to clinical experience from Alpha to Omega.

Dr. Manson, in the course of his public and private practice since March, 1821, has prescribed upwards of 180 ounces of iodine, and, therefore, his experience of its effects is very extensive. He conceives that his report may be productive of some benefit, "by shewing that the prejudices at present entertained against its *internal* use are unfounded; and that it possesses very great powers in the cure of many obstinate diseases, over which other remedies have little or no influence."

I. *Bronchocele.* It is well known that this peculiar disease is very prevalent at Nottingham and its vicinity. Our author, therefore, gave Iodine a fair trial in this complaint, and soon became convinced of its great superiority over burnt sponge in the cure of the disorder. The circumstance of bronchocele being endemic in the author's vicinity naturally led him into some observations on its etiology. The disease is more or less prevalent throughout Derbyshire—but in some places greatly more so than in others. In the village of Cromford, near Matlock, Dr. M. was informed that there are not less than one hundred women who labour under bronchocele of a large size! This proportion is hardly surpassed in Switzerland. The vulgar, in Derbyshire and Nottingham, ascribe the disease "to the hardness of the water."

" This popular notion certainly receives confirmation from the circumstance, that bronchocele is more frequently to be met with, and of a larger size, where the water in common use is very hard, than when it is of a softer quality." 4.

The-well water about Nottingham is more or less hard. In the town itself it is very hard and unfit for washing. Dr. Coindet, in his Observations on Iodine, remarks that "the use of hard or pump water in the lower streets of Geneva brings on the goitre very speedily." This coincides with Dr. Manson's experience. Indeed, there can be very little doubt now that the cause of this strange complaint is to be sought in the waters used by the inhabitants. The following passage, from Franklin's narrative of a Journey to the shore of the Polar Sea, deserves quotation, as recorded by Dr. Richardson.

"'Bronchocele, or Goitre, is a common disorder at Edmonston. I examined several of the individuals afflicted with it, and endeavoured to obtain every information on the subject from the most authentic sources."

"'The following facts may be depended upon: The disorder attacks those only who drink from the *water* of the *river*. It is indeed in its worst state, confined almost entirely to the half-breed women and children, who reside constantly at the fort, and make use of river water, drawn, in Winter, through a hole made in the ice. The men, from being often from home on journies through the plain, when their drink is *melted snow*, are less affected; and if any of them exhibit, during the Winter, some incipient symptoms of the complaint, the annual Summer voyage to the sea coast generally effects a cure. The natives, who confine themselves to *snow water* in the Winter, and drink of the small rivulets which flow through the plains in the Summer, are exempt from the attacks of this disease."

"'A residence of a single year at Edmonston, is sufficient to render a family bronchocelous. Many of the Goitres acquire great size. Burnt sponge has been tried, and found to remove the disease; but an exposure to the same cause immediately produces it.

"'A great proportion of the children of women who have Goitres, are born idiots, with large heads, and the other distinguishing marks of *cretins*. I could not learn whether it was necessary that both parents should have Goitres to produce cretin children.'" 7.

Dr. Manson has not observed any symptom of cretinism accompany bronchocele in this country. As to the particular substance, in solution, which occasions the disease, we are as yet in the dark—"but let us hope that this noxious matter will sooner or later be detected by some one gifted with superior talents for chemical research." We do not know a more important subject of enquiry than this, and we trust that some of our brethren in the goitrous countries will prosecute the investigation.

There appears to be something in predisposition—or, at all events, in sex; for the disease is infinitely more prevalent among women than among men in all countries. The proportion, in Dr. Manson's experience, was 15 males to 101 females. He has known several instances of females who were exempt from bronchocele in their native place, but who became goitrous after a few years' residence in Nottingham. Some of the female children of these parents have also had the disease, at an early period in life, in a still greater degree than the parent—"thereby shewing, in the language of the schools, that the disease is *hereditary*." Although we believe that there are very few constitutional diseases which do not produce some predisposition in the offspring to the same maladies, yet we do not see any proof of hereditary bronchocele in the foregoing fact cited by Dr. Manson. If the female children had bronchocele in a greater degree than their parents, it is to be remembered that *they* were born within the range of the endemic influence, whereas their parents did not come within this range till they were grown up. This must make a great difference.

"In almost every case when the Thyroid gland is enlarged in a considerable degree, more or less hoarseness, difficulty of breathing, and wheezing take place, when the patient walks fast, ascends a stair, or runs. But I have found the greatest degree of dyspnœa and wheezing to take place in those cases in which the middle portion of the gland is developed in a globular form, and, by pressing on the trachea, occasions much distress in breathing." 10.

Our author has met with only three instances of bony tumour in the enlarged thyroid gland. Previously to the discovery of iodine as a remedy, our author used burnt sponge and alcaline preparations, with the usual success; but when Dr. Coindet announced the effects of iodine, Dr. M. prepared a formula, consisting of one drachm of iodine to two ounces and a half of rectified spirit, (spec. gr. .916) which is just half the strength of Coindet's mixture. Dr. Manson has presented a tabular view of 116 cases of bronchocele treated by this remedy, and also the *details* of 15 other cases, with remarks. A series of remarks are also made on the more interesting cases in the table. We may premise that, of the above tincture, our author appears to have given from 10 to 30 drops thrice a day, according to the age and strength of the patient. It may also be proper to state, that one of the patients, (No 18 in the table) a young woman, swallowed, by mistake, nearly an ounce of the tincture of iodine, without dilution. It was immediately rejected by vomiting, and no inconvenience or bad effects ensued. We can only give the result of the table of 116 cases, recommending both it and the detail of 15 other cases to the serious attention of the profession.

| | | | | |
|---|---|---|---|---|
| Total number of Cases | | | | 116 |
| Viz.—Males—— | Cured | 10 | | |
| | Much relieved | 1 | | |
| | Discharged for non-attendance | 1 | | |
| | Improving under treatment | 3 | —Total 15 | |
| Females— | Cured | 66 | | |
| | Much relieved | 9 | | |
| | Not relieved | 2 | | |
| | Discharged for non-attendance | 10 | | |
| | Improving under treatment | 14 | —Total 101— | 116 |

Dr. Manson did not confine his practice to the internal exhibition only of the remedy. He occasionally used a liniment, composed of liniment. sap. comp. ℥j. tinct. iodini ʒj. misce, ft. linimentum. Most patients can bear this quantity to be rubbed into the tumour once, or even twice a day. In some persons, however, the skin is so tender, that the liniment must be less frequently used. The liniment abovementioned Dr. M. considers as a very useful form for the external administration of iodine, having this advantage over the iodine ointment, that evaporation can be prevented by keeping it in a stopped vial.

"In *some individuals*, after the preparations of Iodine have been given internally for some time, they are apt to occasion head-ache, giddiness, sickness at stomach, with some degree of nausea, languor, and inaptitude for exertion; when these unpleasant sensations and effects occur, the best plan to remove or obviate them is, to suspend, for a time, the use of the medicine, or to reduce the dose, as may seem most expedient. A reduction of dose, from fifteen to twelve drops, was the plan adopted by me on this occasion." 65.

We were happy to observe, by the following extract, that, in the hands of Dr. Manson at least, the internal use of iodine was not productive of any serious or lasting bad consequences.

"As I should be considered tedious by offering more remarks on individual cases of Bronchocele, I may now generally observe, that in the other cases, so far as I had an opportunity afforded me of following up the treatment by the internal exhibition of Iodine, they all went on favourably, without any particular *unpleasant effect* from the medicine, and without any *injury* to the *constitution*. On the contrary, the Tincture of Iodine, and other preparations of it, employed by myself, had generally a cordial and tonic effect, unless given in too great a dose; and the patients to whom it was exhibited, with the exception of the persons mentioned in the remarks, generally found themselves in better health and spirits than they had previously been for years. This observation applies, not only to those who laboured under Bronchocele, but also, as will appear from the sequel, to those who laboured under other diseases, in which Iodine was exhibited.

"It is a great satisfaction for me to be able to state, that the patient (No. 30) with whom the Iodine *disagreed* the most, and who derived the *least benefit* from it, has *again* put herself under my care (see No. 112,) and is taking small doses of the Hydriodate of Potass, and using the Linimentum Iodini externally, not only without inconvenience, but with some advantage. I think it necessary to point to the above circumstance in a particular manner, as it appears from the report of a celebrated author and reviewer, that Dr. Coindet now considers the *internal* exhibition of Iodine as injurious, and uses it only externally, in the way of friction. I apprehend that Dr. Coindet has exhibited this powerful medicine in too large doses for the generality of patients, otherwise he could have been under no alarm about its internal use.

"The rule to be observed with Iodine, as with all other powerful medicines, is to begin with a small dose at first, and to increase it cautiously, and at proper intervals; in this way we shall avoid doing harm, and shall be able to discover any peculiarity of constitution before the dose of the medicine can amount to a quantity sufficient to injure the constitution, or even to occasion much inconvenience.

"The cases and the table will shew the doses of the different preparations of Iodine employed by me, as well as the ages of the patients, &c. I beg particularly to observe, that it is absolutely necessary to keep the bowels in an *open* and *regular state* during the exhibition of Iodine.

"The power of Iodine over the absorbent system, has been noticed by Dr. Coindet and others: it early attracted my attention, as will be seen by referring to the Remarks, No. 18; since that time, as will afterwards appear, I have had ample proof of its great power over the lymphatic system; but I am of opinion that it exerts no peculiar or specific power over that system of vessels and glands, but that they only participate in its general energetic effects on the whole body.

"I cannot suppose that a temporary excitement given to the absorbents, would remove large Bronchoceles, and prevent their return, unless the state of the system that gives rise to goitre was corrected, when that is accomplished, the absorbents perform their office with effect, and the swelling disappears; but, in my opinion, it is incorrect to ascribe the *whole* cure to increased absorption.

"Before concluding my remarks, and bringing to a conclusion what I have to communicate on the subject of Iodine, I can, without hesitation, state, that I have found it a much more convenient and effectual remedy, in dissipating goiterous swellings, than burnt sponge, which I had employed, very frequently with success, for nine years before I knew any thing of the remedial powers of Iodine. Independent of burnt sponge being a bulky and unpleasant article to take, it is very clear that we never can know the quantity of Iodine contained in any given quantity of this medicine, or even whether it contains any Iodine or not; burnt sponge must, therefore, be considered as a very uncertain remedy, compared with Iodine, and will, I have no doubt, soon give place to it.

"In confirmation of the beneficial effects of Iodine in the cure of Bronchocele, I may state, that Mr. Jowett, a highly intelligent young Surgeon, who has for some time performed the duty of resident Medical Surgeon at Saint Mary's Hospital and Dispensary, of this town, lately informed me, that he has cured two cases of Bronchocele by the internal exhibition of the Tincture of Iodine, without inconvenience to the patients, or any injury to the general health." 68.

II. *Paralysis.* The different species of palsy occur so frequently, and are so difficult to cure, that any new remedy which strengthens our hands in such complaints, will be received with gratitude by the medical world. It was want of success with the usual remedies in many cases of palsy, that led our author to try a new one in a very deplorable case, of which we shall present the reader with some particulars presently.

"The wonderful powers of Iodine, which I had recently witnessed, and a long previous acquaintance with the effects of the same remedy, as it exists in burnt sponge, in reducing morbid enlargements of the Thyroid gland, led me, from analogy, to think, that in cases of palsy, from tumours or fluids pressing on the brain or spinal cord, or from morbid thickening of the investing membrane of the cord itself, Iodine might prove a useful remedy, not only by stimulating the nervous system, and removing morbid tumefaction and effusion, but also by correcting the strumous state of the constitution that often gives rise to the disease." 74.

*Case of Paralysis.* J. Watterton, aged 19, was admitted into the General Hospital of Nottingham on the 27th of March, 1821, having been ailing since October, 1819. Stated that he had at first been attacked with pain in the bowels, which pain having ceased, the lower extremities became swelled and painful. After this his neck became stiff and painful, with shooting pains from the neck into the left side of the head. These also disappeared, and did not afterwards return. This was about nine months ago, and, at that time, he suddenly lost the power of the left arm, and, in a short time afterwards, that of the left lower extremity. Some time after this he recovered, partially, the use of the left arm, the leg remaining paralytic. About this time, the *right* half of the body was instantaneously and completely palsied. He has continued ever since in this wretched state, getting worse rather than better, passing his stools and urine involuntarily. He lies on his back, and, with the exception of the left arm, he is completely paralytic on both sides, from the neck downwards. The sense of feeling is very much impaired—there is no distortion of the face, nor impediment of speech. Is troubled with twitchings in the lower extremities. *Purgatives—blister to the nape of the neck, and to be kept open.*

It appears that, about two years ago, he had a bloody purulent discharge from both ears. The left ear still continues to discharge a purulent-looking matter. Purgatives were continued till the 6th of April, when the tincture of iodine, in doses of 15 drops, was given thrice a day. April 9, can raise the right arm nearly to his head; but the power of the lower limbs has not improved. The twitchings have decreased. Purgatives—the tincture of iodine to be increased to 20 drops ter in die. 10th. Evinces some muscular power in the lower extremities to-day—feels stronger—can retain his urine for some time. 14th. Continues to improve. The left foot is become exquisitely sensible, and that extremity is often drawn up spasmodically towards the body. The iodine to be increased to 25 drops. 16th. "The paralytic symptoms continue to yield to the powerful influence of the iodine." When his meat is cut, he can now feed himself with the left hand:—can raise the right hand to the chin, and draw the right lower extremity up towards the body. He continues to hold his water. The iodine in doses of 30 drops ter in die! Passing over minute details, which shew that the medicine was obliged to be intermitted occasionally, and again commenced in smaller doses, we find, on the 7th of May, that the patient can walk from his bed-room to the day-ward, with very little assistance. 19th. He can walk without any assistance, except that of a stick to steady him. *June 9th.* Can walk without a stick. "He is gradually recovering the power of motion and sense of feeling. Drops agree. Appetite good, and is now allowed full diet." *July 3rd.* The patient was discharged, cured.

This is a very interesting case, and the result must have been extremely gratifying to the physician, as well as the patient.

"I think it necessary to remark, that after taking thirty drops of Tincture of Iodine thrice a day, for five days, he was attacked with head-ache, and his pulse rose to 96 beats in a minute. The Iodine was omitted—the head-ache went off, and in a few days he was ordered thirty drops of the Tincture of Iodine as before, to ascertain whether the head-ache, and other febrile symptoms, were occasioned by the Iodine, or owing to some other cause. In three days after resuming the use of the Iodine, the head-ache was as severe as ever, and the pulse up at 100. I was now convinced that the dose of Iodine was too large, and, after omitting it for two days, I ordered 15 drops three times a day, and gradually increased the number of drops, so that they did not afterwards disagree with the patient. From the history of the case, the purulent discharge from the ears, the swelling and stiffness in the neck, and the general appearance of the patient, I have no doubt that the Palsy, in this case, was owing to scrofulous inflammation, tumefaction, and, perhaps, effusion, by which the spinal cord in the neck was so compressed as to render the parts below it paralytic. But, whatever pathological opinion may be formed of this case, the facts must still remain the same." 89.

Several other cases are related by our author, but none of them quite so striking as the foregoing one. In some instances of hemiplegia, however, the influence of the medicine was very satisfactory; and, upon the whole, the evidence of its salutary operation in this deplorable complaint is so unequivocal, as to authorize practitioners to have immediate and extensive recourse to the medicine.

We should have observed that Dr. M. has given several very interesting dissections of patients dying of paralytic affections, in which researches he was ably assisted by Mr. Jowett, a young gentleman who is likely to distinguish himself in his profession by a happy combination of zeal and talent. In this place, Dr. M. recommends an easy mode of opening the spinal canal, "by striking each vertebra with a plumber's 'hacking' knife and a hammer, about midway between the spinous process and transverse process on each side. After the first portion of vertebra is got out, it is really surprising with what rapidity the whole canal may be brought into view."

III. *Chorea.* The benefit derived from the use of iodine in palsy, induced Dr. M. to try its effects in chorea—" a disease more nearly allied to palsy than is generally supposed, and of which shaking palsy seems to form the connecting link." To shew that he is not singular in this opinion, our author quotes passages from Drs. Good, Sydenham, Cullen, Hamilton, &c. but, as Dr. M. remarks, it is perhaps a matter of very little consequence whether chorea be considered as a species of palsy, or stand as an independent genus.

"The involuntary motions in chorea are considered as convulsions by Sydenham and others; to me they have never appeared to resemble convulsions, such, at least, as we observe in Epilepsy; but seem more like the random action of the muscles, when the power of the will over them has been partially or wholly suspended, so that it has no longer any proper controul over the motions of the parts affected, although it may have sufficient influence to originate them." 183.

This curious disease is much more prevalent among females than among males. During the last twelve years, 76 cases were treated at the Nottingham General Hospital, of which 22 were males and 54 females. Dr. M. observes, that although the profession are much indebted to Dr. Hamilton for drawing their attention to the generally disordered state of the primæ viæ in chorea, yet, when he reflected that the sufferers under this disease are—"those chiefly (using Dr. Hamilton's words) who are of a weak constitution, and whose natural good health and vigour have been impaired by confinement, or by the use of scanty or improper nourishment," he (Dr. M.) has generally been deterred from the vigorous exhibition of purgative medicines in this disease, and has confined himself to the exhibition of purgatives in the first place, to unload the bowels, and afterwards to keep up a regular alvine discharge, avoiding the debility occasioned by full purging, as far as possible. After the bowels have been unloaded, and the regularity of their action established, our author has exhibited bitter vegetable tonics, with general success—at least in the milder cases; but in the severe and protracted forms of the disease, the vegetable tonics occasionally lose their effects from long-continued use. This circumstance, which, indeed, applies to all medicines, renders it desirable that we should possess two or three remedies capable of curing a disease, so that when one is disliked, or ceases to have a beneficial effect, we may be able to employ another in its stead.

Our author was prepossessed with a favourable opinion of iodine, as a remedy in chorea, from its stimulating qualities, tonic effects, and the diffusive energy which it exerts on every part of the body. Eleven cases of chorea are detailed by our author, all shewing the efficacy of iodine, administered after purgatives, and while the bowels were carefully regulated by aperient medicines. The first case is very instructive.

A young girl, a lace-runner, had laboured three months under chorea when she came under Dr. Manson's care. She was briskly purged for nearly a month, and although her power of locomotion improved, the involuntary action of the muscles continued, and the impediment of speech. Our author then gave the carbonate of iron, in ten grain doses, thrice a day, administering purgatives occasionally. This plan succeeded much better. In a fortnight the involuntary motions had diminished very much, and she could articulate much better. Nevertheless the disease shifted to the other side of the body, and the business was all again to be gone over. The steel and purgatives were again tried, but failed. Then the oxyd of zinc was exhibited, and it also failed. In this extremity the iodine was administered in the usual manner, and the disease was soon entirely extirpated. This section concludes with a tabular view of 72 cases treated at the Nottingham Hospital before alluded to.

IV. *Scrophula.* Dr. M. considers this as a disease of debility—and the disposition to it as hereditary. But we meet with it as a primary affection, and where we are unable to trace it to any ancestor.

"The exciting causes of scrophula in the predisposed, are want of proper exercise, living in impure air, inhabiting cold damp houses, using unwholesome food, or having too scanty a supply of what is proper, disorder of the bowels, of considerable duration, atmospherical vicissitudes, particularly a long continuance of cold moist weather, commonly called raw, after fine and warm weather, especially when the person is deficient in warm comfortable clothing." 233.

It is hardly necessary to notice the different plans of treatment that have been proposed, since we may even now repeat what Cullen said long ago—"for the cure of scrofula we have not yet learnt any practice that is certainly, or even generally, successful."

Dr. Manson details three cases of scrofulous enlargement of the conglobate glands—two cases of scrofulous ulcers—and four cases of scrofulous ophthalmia, treated by iodine, and with decidedly beneficial effects.

"The above cases," says Dr. M. "are, I conceive, sufficient to shew the great efficacy of iodine exhibited internally, as a remedy in scrophula, and that it possesses the wonderful powers, when cautiously exhibited, of correcting and improving strumous constitutions. This, I believe, is partly owing to its stimulant and tonic powers, but the whole of my experience with this potent medicine, leads me to believe, that it also possesses the remarkable power of correcting morbid action in many diseases by which the human body is assailed, analogous to that of mercury in syphilis; and I have no doubt, that the great success I have had in the treatment of palsy and chorea, by this new remedy, has been, in a great measure, owing to its correcting morbid action in the brain and spinal marrow, at the same time that its other qualities come in aid of this highly valuable property." 257.

V. *Fistula Lachrymalis.* A patient of Dr. Manson's, who was taking iodine for paralysis, happened to labour under fistula lachrymalis. The improvement in the latter complaint was of so marked a character, in every respect, that our author availed himself of every case of the disease that afterwards came under his care, with the view of ascertaining the power of iodine in its cure;—"and every additional case confirms me more and more in the firm belief of the great remedial powers of iodine in this untractable disease." Dr. M. then goes on to detail eleven cases, one of which we shall here quote entire, as a specimen.

"*Case.* Miss Ann P, æt. 19. December 23, 1822. This young woman is afflicted with bronchocele of moderate size,* and for the last eight years has laboured under obstruction of the left lachrymal duct, for which I find she was operated on by the late Mr. Stanley, Surgeon, of this town. The lachrymal sac is considerably swelled, and when it is pressed with the finger, a copious flow of thin mucus, and some purulent-looking matter is discharged through the puncta lachrymalia into the left eye. The lachrymal sac has inflamed, swelled, and burst nine or ten times; it is about two months since this last occurred. The opening has healed up, and she says that she is obliged to squeeze the contents of the sac out twelve or fourteen times a day, to prevent the inconvenience of its swelling and bursting. The left eye is almost constantly filled with tears, which affect the vision, and often flow down the cheek in great quantity, and she says, that the complaint makes her altogether very uncomfortable. Complexion fresh. Eyes and hair black. Bowels, in general, regular. General health not to be complained of. Catamenia regular.

"Sumat Tæ. iodini gutt. xii. ter in die ex cyatho parvo aquæ. Instillr. guttæ vi. Vini opii in oculo sinistro m. et h. s.

"January 1, 1823. Her eye is not so full of tears, and thinks that, since yesterday, some of the tears have passed by the nose, and she can squeeze but little mucus from the lachrymal sac into the eye, compared to the quantity that was poured through the puncta lachrymalia when I first saw her. Medicines agree very well.

"Capiat tæ. iodini gutt. xv. ter in die. Contr. alia.

"January 19. I have visited this patient about twice a week since last report. The eye waters much less, although the weather is colder than it was when she became my patient. The discharge through the lachrymal duct into the nose increases, and she says that she seldom has occasion now to press on the lachrymal sac to discharge its contents, which still partly pass into the eye. The tincture of iodine sometimes makes her sick, but she never rejects it by vomiting, The bronchocele is nearly dissipated.

"Contr. remed.

"February 18. Her eye waters very little, the swelling of the lachrymal sac has subsided, and the nasal duct is pervious to the lachrymal discharge. Says that she feels an improvement in her general health.

"Contr. remed.

---

" * See Table, No. 54."

"March 7. The left eye scarcely waters at all, and there is very little discharge into the eye when pressure is made on the sac. The nasal duct remains pervious. She looks and feels better.

"Contr. remed.

"March 15. Eye waters very little, there is no return of swelling in the sac, and it has not burst since she began to take the tincture of iodine. The nasal duct remains pervious to the lachrymal discharge. She is desired to employ the medicines a few weeks longer, by way of confirming the cure.

"*Remarks*, From the chronic and stubborn nature of fistula lachrymalis, it is impossible for the most sceptical, on any fair grounds, to question the medical agency of the tincture of iodine in this instance, as the cure was not only soon accomplished, but the patient has remained well ever since, without any tumefaction or bursting of the sac, and her eye seldom waters, unless she accidentally takes cold.

"It deserves notice, that she has lost one sister of consumption, another is afflicted with lepra vulgaris, and a third, who is married, resides at Derby, and labours, like my patient, under fistula lachrymalis." 278.

If iodine proves as successful in this unsightly deformity, in the hands of others, it will be a feather in the cap of physic, and curtail the use of the knife. We sincerely hope it may prove a substitute for this last weapon, not only in fistula lachrymalis, but in white swellings of the joints, whereby some limbs may be occasionally saved to His Majesty's subjects.

VI. *Deafness.* Next to the loss of sight is that of hearing. Indeed it is generally remarked that blind people are much more cheerful than those who are entirely deaf. The small progress that has hitherto been made in the cure of deafness has induced our author to lay before the profession a series of cases (nine in number) "in which the effects of iodine, as a remedy for this obstinate malady, were very conspicuous." The causes of deafness must be very various, according to the part of the complex organ of hearing which happens to be affected. Deafness, from obstruction of the eustachian tube, whereby air is prevented from getting into the internal ear, appears to be a very common occurrence, and it is probable that it is in such cases that iodine is most useful.

"When a considerable degree of tumefaction of the soft parts of the external passage occasions deafness, and leeching and blistering have been of no avail, I think it probable that iodine administered internally, and applied externally, in the form of liniment, or ointment, before and behind the ear, in the vicinity of the passage, would be found useful in reducing the morbid tumefaction of the parts, and in contributing to the improvement or restoration of hearing." 307.

Our author discovered the remedial powers of iodine in the cure of deafness, when treating a patient who laboured under bronchocele, and the unequivocal benefit resulting in this case, induced him to try it in many others.

"The remarkable powers of this medicine, in arresting morbid action, and in reducing the unnatural enlargements of the soft parts of the body, point it out as the appropriate remedy to remove the tumefaction of the soft parts at the termination of the eustachian tube, in consequence of which the function of the organ is suspended. The cases about to be detailed will shew that iodine possesses very great remedial powers in curing deafness, arising from obstruction to the passage of air to the internal ear, and I hope it will, in a great measure, supersede the necessity of puncturing the tympanum, in this variety of the disease." 309.

Dr. Manson properly observes, that the cautious trial of this medicine in deafness arising from disorder of the internal ear, is not to preclude the employment of leeching, blistering, syringing, and other means that may be judged necessary in such cases. We shall here, as in the preceding section, introduce a single case as an example.

"*Case.* Mary Wood, æt. 16, a servant, from North Wingfield, Derbyshire. Applied to me the 8th of October, 1823, on account of a considerable degree of deafness under which she has laboured since she had the scarlet fever, about eleven years ago. Has had a purulent discharge from the right ear for the same period, and the left ear also discharged matter, till about a year since. Says that she had gatherings under the lower jaw, when a child, and she has some scars in the same situation, that mark the place where conglobate glands have suppurated and burst, at some former period. Complexion florid, hair light brown. Her general health is pretty good now, and she has menstruated regularly for the last six months. Bowels, in general, regular. Her hearing is always worse after taking cold.

"Capiat tæ. iodini guttas x. ter in die ex aquæ cyatho, et ℥ij magnesiæ sulphatis alternis matutinis.

"October 11. Thinks she can hear a little better already.

"Contr. remed.

"October 18. Her hearing is evidently better, as appears by her knowing what is said in a lower tone than when I first saw her, and the patient herself is sensible of the improvement. Bowels open by the Salts.

"Capiat tæ. iodini gutt. xv. ter in die.

"November 8. Continues gradually to recover her hearing. Medicines agree.

"Contr. remed.

"This patient continued the use of the Tincture of Iodine till she had taken three half ounces of it in doses of fifteen drops, three times a day, and did not take any more of it, her hearing being perfectly restored.—Cured.

"REMARKS.—I find that I have neglected to note down the time, in my case book, when the cure was completed, but it was after she had taken the medicine in the quantity stated above. I called to see this young woman the 4th of February, 1825, and I have the satisfaction to add, that she has grown both taller and stouter, and that her hearing continues perfect." 319.

Dr. M. has several interesting cases of deafness under treatment at present, but they are not in a state of forwardness for a report.

VII. *Dysphagia.* This complaint, like deafness, arises from various causes, but the observations which Dr. Manson has to detail, relate to difficulty of swallowing from a permanently contracted state of the œsophagus, preventing the free passage of food into the stomach. Strictures of this tube are most frequently found near its upper extremity, and, next to that, near the cardia. Dr. M. was first induced to exhibit iodine in this disease, from having witnessed its great powers in reducing swellings of the thyroid gland, " and which I could not account for, as some have done, from mere increased absorption but was convinced that it possessed, in an eminent degree, the power of suspending and removing the morbid action on which the disease depended, otherwise it would soon have returned." Under the influence of this reasoning, Dr. M. prescribed the tincture of iodine in the following case, and with the most complete success. This case we shall select for quotation here.

"Mrs. ———, æt. 45, tall and stout made, eyes and hair black, complexion dark. Was on a visit to a family near this town, where I was in attendance, the latter end of April, 1821, and consulted me respecting a difficulty in swallowing, attended with some pain at the top of the gullet, with which she had been troubled for three years preceding. She informed me, that she had had the assistance of the family surgeon (an excellent practitioner, and many years an hospital surgeon) who passed a bougie, about the size of her ring finger, four times into the gullet. The first time it occasioned a good deal of pain, in passing the strait part, and the last time occasioned some discharge of blood, but gave less pain than the first time it was introduced into the œsophagus. The patient swallowed a little better, for a time, after each introduction of the bougie; but when she applied to me, she thought the complaint upon the whole gradually becoming worse, and could only swallow fluid, or very soft food, as mashed potatoes, with a little gravy, when she requested my assistance. She also laboured under a degree of bronchocele, but that tumour had no share in producing the dysphagia, which I am of opinion, was occasioned by a thickening of the coats of the œsophagus, and the surrounding parts, which Sir E. Home says, 'in the end generally becomes cancerous; or, in other words, an incurable disease.' The time of life of the patient also corroborates the view I took of the case, as the catamenial discharge had become irregular. In revolving all the circumstances in my mind, I could think of no medicine, in ordinary employment, that was likely to be of any lasting service; but it occurred to me, from analogy, that iodine was more likely to be of service in this formidable case, than any article of the materia medica, from its singular and highly valuable property of subduing morbid action, and removing tumefaction at the same time. I therefore conceived myself not only justified, but called upon to give the patient the chance of the benefit that might result from a cautious employment of this active substance. I may observe, that the patient's general health at the time, was but indifferent, and she was usually troubled with a costive state of the bowels. The following medicines were prescribed:

"℞ Pilæ. cambogiæ comp. ʒii forma in pilulas xxiv. quarum sumat ii vel. iii. omni nocte si adstricta est alvus.

"℞ Tæ. iodini ℥ss., capiat ægra guttas x. ter in die ex cyatho vinario aquæ, et post septimanam sumat gutt. xv. ter in die.

"About a fortnight afterwards, I called upon the patient, at the house of a relation, in this town, and found that she could swallow with more ease, and that the medicine agreed very well with her. Bowels kept open by two or three pills.

"As the patient was obliged to go into Leicestershire, and intended remaining there for some time, I gave her general directions how to take the iodine and the aperient pills. I learnt from mutual friends, that she had perfectly recovered before I had the pleasure of meeting her in January 1825, at the same house where I first prescribed for her; when I learnt from herself, that the medicine had been of the greatest service to her; that she had gradually increased the dose of the drops, till they amounted to twenty-five, three times a day; and at the end of five months she was perfectly free from any difficulty in swallowing, and found her general health greatly improved. She informs me, that she has continued to enjoy excellent health ever since, and can swallow liquids and solids as well as she ever could in any former period of her life. I found she took about two ounces and a half of the tincture of iodine, before finally leaving it off, but she has continued the aperient pills, occasionally, ever since.

"REMARKS. The leading facts of this case are well known to several highly respectable individuals in this town and neighbourhood, and the lady herself, who now resides about thirteen miles off, will, I trust, long remain a living witness of the utility of iodine in the cure of dysphagia." 333.

8. *White Swelling.* The benefit which our author has seen derived from the internal exhibition of iodine in this formidable disease, justifies, he thinks, the most sanguine expectations from its powers as a constitutional remedy, if early exhibited and steadily persevered in. At the same time, we are not precluded from the employment of any topical means that may be deemed proper or necessary, as auxiliaries in so obstinate and untractable a disease.—Eleven cases are detailed by Dr. Manson, and although our limits are fast closing on us, we must endeavour to make room for one of these cases, as a specimen.

"*Case* XI. Mr. ———, æt. 47, a master stone mason. August 1 1823. There is an uniform swelling of the left knee, which gives him pain when he walks; and when he is seated, and attempts raising the leg into a horizontal posture, the pain darting through the affected joint is so great, that he is instantly obliged to desist. The skin of the knee is not in the least discoloured, but the veins are swollen, and appear, from that circumstance, to be more numerous than natural. The patella is not to be discovered by the eye, the ham is fuller than natural, the left leg and thigh are wasted, in a certain degree, and the knee measures an inch more than the right. The right ankle has been swelled and stiff for the last three months, and when he walks it gives him pain. The affection appears to be of a similar nature to that of the knee. About four or five years ago, I find he was under a quack for the cure of bubo; the gland did not suppurate, it still remains larger and harder than natural, but is free from soreness. Is subject to gravel, and has at times passed small calculi, about the size of common shot, for the last ten or twelve years. Pulse 96, and of moderate strength. Tongue a little furred. Appetite moderate. Was under the prescription of two eminent physicians, of this town, in succession, for a considerable time, but without any benefit. He next applied to the late Dr. W. of Ripon, in Yorkshire, but without relief. He afterwards put himself under the care of Mr. J. whom he represents as an eminent surgeon, in Yorkshire, and he thinks that this gentleman was of more service to him than any other practitioner; but as he failed in doing more than affording temporary relief, he afterwards applied to different irregular practitioners, some of whom, he informs me, made use of severe applications to the knee, but without any permanent benefit, and others of the same fraternity would not meddle with the case, as being beyond their skill. I know nothing of the means made use of by the different parties in this case; and if I did, it would be of no use to enumerate them, as it does not appear, from the patient's statement, that he derived any lasting benefit from any of them.

Capiat Pil. Hydrarg. gr. x. m. et h. s. Sumat Tæ. Iodini gutt. xv. ter in die ex aquæ cyatho.

℞ Adipis Præp. . . . . . . . . . . . . . . . . . . . . . . . . ℥i.

Tæ. Iodini. . . . . . . . . . . . . . . . . . . . . . . . . . . ʒiss.

"Misce, ft. Ung. cum quo optimè inungatur genu affectum mane et horâ somni. Utat$^{r.}$ Balneo ad gradum 96$^{um.}$ bis in septimana.

"August 6. Feels much better. The left knee measures nearly the same as the right. The patient remarks, that the swelling is so much gone, that he now can see the knee pan. Says that the pain is very much abated, and that he can walk better.

"Cont$^{r.}$ remed.

"August 22. I have visited the patient about twice a week, since last report. After the pain and swelling in the knee abated, he began to complain of pain in the long bones; and the left testis began to swell about the same time, but the tumefaction was not attended with acute pain. The same treatment has been continued, except that he has taken twenty drops of the tincture of iodine for a dose. To-day he can raise the left leg, and support it in the horizontal posture, without any inconvenience, which he says he could not have done for the world when he came to me. The right ankle joint is also much easier, and the swelling greatly reduced. The pains in the middle of the long bones are easier, and he is altogether so much better and easier, that he has lately attended to business, and even has done some light work himself. He has used an alum gargle to prevent his mouth from becoming sore. The right ankle has been rubbed with the iodine liniment for the last fortnight.

"Cont$^{r.}$ remed.

"August 29. Continues to improve, and complains of very little pain. The left testis is still swelled, but not painful. Mouth not sore.

"Cont$^{r.}$ remed.

"September 4. He complains of very little pain in the knee, even when he walks, and when at rest is perfectly free from it. When he stands or walks, he still feels a little pain in the right ankle joint, which is scarcely more swelled than the left. Was purged five or six times yesterday, and he has had three motions, with some tenesmus, to-day.

"Cont$^{r.}$ remed. Sumat Tæ. Opii gutt. xv. h. s. et gutt. x. bis dè die perstante diarrhoeâ.

"September 10. The patient continues to improve in health, and to recover flesh and strength. He works all day. Left knee no larger than the right, and it is free from stiffness, but feels a little weak. He says that the right ankle is nearly well. Left testis still a good deal swelled, but it is free from pain. The purging was soon stopped by the tincture of opium.

"Cont$^{r.}$ remed.

"October 4. Is greatly improved; indeed, he says that he feels nothing the matter with him.

"Cont$^{r.}$ remed.

"No more notes of the case were taken after the 4th of October. The patient continued to take the medicines with great regularity till Christmas, 1823, with the exception of fourteen days, during which time they were omitted by my direction. When he left off the medicines at Christmas, he felt very well, and has enjoyed excellent health and spirits ever since." 387.

The two remaining sections on "morbus coxarius," and "distortions of the spine," we must pass over, as we have exceeded the space we had allotted for this analysis. We are quite convinced that what we have extracted from the work will lead to a general perusal of the original, and, we sincerely hope, to a considerable mitigation of human suffering, through the agency of the powerful medicine so extensively employed by Dr. Manson. This gentleman deserves the thanks of his brethren for laying before them such a large mass of authentic facts and unostentatious observations.

